# Plasma Kisspeptin Levels in Newborn Infants with Breast Enlargement

**DOI:** 10.4274/jcrpe.1994

**Published:** 2015-08-31

**Authors:** Avni Kaya, Zerrin Orbak, Harun Polat, Atilla Çayır, Abdullah Erdil, Hakan Döneray

**Affiliations:** 1 Atatürk University Faculty of Medicine, Department of Pediatric Endocrionology, Erzurum, Turkey; 2 Atatürk University Faculty of Medicine, Department of Biochemistry, Erzurum, Turkey

**Keywords:** Kisspeptin, newborn, breast enlargement

## Abstract

**Objective:**

Kisspeptin levels have been reported in children with premature thelarche, precocious puberty and adolescent gynecomastia, but there are no reports on kisspeptin levels in the neonatal period. This study aimed to investigate plasma kisspeptin hormone levels in newborns with and without breast enlargement.

**Methods:**

Plasma kisspeptin levels and other related biochemical variables were investigated in this prospective study conducted on 40 (20 girls and 20 boys) newborn infants with breast enlargement and on 40 healthy control infants (20 girls and 20 boys). Two-milliliter venous blood samples were taken in hemogram tubes with K2EDTA. Kisspeptin assays were performed using the enzyme-immunoassay method.

**Results:**

Mean plasma kisspeptin levels were 0.6±0.2 ng/mL in the study group and 0.5±0.2 ng/mL in the control group. Plasma kisspeptin concentrations were significantly higher in the study group (p=0.039) and also showed a correlation with serum prolactin levels (p=0.006). Significant correlations were also determined between plasma kisspeptin and luteinizing hormone concentrations (p=0.05, r=0.312).

**Conclusion:**

The findings of this study suggest that plasma kisspeptin and serum prolactin levels may be involved in the physiopathology of breast enlargement in newborns.

## INTRODUCTION

Kisspeptin is a powerful neuropeptide that stimulates the release of follicle-stimulating hormone (FSH) and luteinizing hormone (LH) from the pituitary. It exhibits this effect through the gonadotropin-releasing hormone. Kisspeptin is synthesized in the anteroventral periventricular nucleus and the arcuate nucleus of the hypothalamus (1). A significant rise in FSH, LH and testosterone levels when kisspeptin is administered intravenously demonstrates its role in the hypothalamo-pituitary-gonadal axis (2). Kisspeptin is one of the peptides regulating the neuroendocrine events initiating puberty in humans and animals (3,4,5). Kisspeptin levels have previously been studied in premature thelarche, precocious puberty and in gynecomastia in adolescence, but to our knowledge, there are no reported studies on kisspeptin in breast enlargement in the neonate (6,7,8).

The aim of this study was to determine and compare kisspeptin levels in 14-28-day-old infants with and without breast enlargement.

## METHODS

This prospective study was performed on infants with and without breast enlargement who presented to the Pediatric Endocrinology Outpatient Clinic of the Atatürk University Faculty of Medicine Research Hospital in Erzurum, Turkey, between September 2013 and March 2014. The purpose and design of the study was explained to all the families and the requisite written consent was obtained.

Forty term infants (20 boys and 20 girls) aged 14-28 days with breast enlargement and 40 (20 boys and 20 girls) control infants without breast enlargement and of the same age group were included in the study. Criteria for exclusion consisted of presence of congenital abnormality in the infant and/or chronic disease in the mother. Breast enlargement was evaluated on the basis of the breast diameter. Infants who showed breast enlargement of more than 1 cm in diameter were included in the study. Infants without breast enlargement constituted the control group.

Following receipt of informed consent, prenatal and postnatal histories including week of gestation at birth, age, type of delivery (cesarean section delivery or vaginal delivery) and type of feeding (mother’s milk and/or formula) were taken from the mothers and physical examination was conducted on the infant. The infant’s age, length, body weight, breast stage and testis volume (using a Prader orchidometer) were recorded. Apart from breast enlargement, physical examination was normal in all infants. Blood samples were collected from all babies for measurement of FSH, LH, estradiol, testosterone, prolactin, free thyroxine (fT4) and thyroid-stimulating hormone (TSH). Kisspeptin levels were determined on blood samples which were collected from the infants with breast enlargement and controls in K EDTA hemogram tubes and centrifuged at +4 0C to separate blood cells and plasma. These were placed in Eppendorf tubes and stored at -80 0C until the acquisition of kisspeptin kits (KiSS-1 (112-121) Amide/Kisspeptin-10/Metastin (45-54) Amide (Human) EIA KIT, Phoenix Pharmaceuticals Inc., USA, Catalog No: EK-048-56, Lot No: 604601). Plasma kisspeptin levels were measured, following the manufacturer’s instructions, using the enzyme-immune assay (EIA) technique at the Atatürk University Hospital Department of Biochemistry. Kisspeptin levels were expressed as nanograms/milliliter.

Numerical data were expressed as minimum, maximum, mean and standard deviation. Other data were expressed as percentages. SSPE 20 for Windows was used for data analysis. The one-sample Kolmogorov-Smirnov test was used to determine whether numerical data in the thelarche and control groups were normally distributed.

The infants’ body weight, length, postnatal ages, body mass index, LH, testosterone, prolactin, fT4, TSH and kisspeptin levels were normally distributed according to the one-sample Kolmogorov-Smirnov test. The independent-sample t-test was used to analyze these data. FSH, estradiol levels and FSH/LH ratio were non-normally distributed according to the one-sample Kolmogorov-Smirnov test. The nonparametric two-independent-samples Mann-Whitney U test was used to analyze these data. Type of delivery, feeding status and testis size were determined using the Pearson’s chi-square test. Statistical significance was considered at p<0.05.

The study was approved by the local ethics committee (Atatürk University Faculty of Medicine Ethics Committee on September 2, 2013, Session 6, Item 62).

## RESULTS

Mean age of the cases in the breast enlargement group was 20.6±4.1 days (14-27 days). Mean body weight was 4.1±0.6 kg (3.3-5.8 kg) and length 52±1.7 cm (49-57 cm). Twenty-nine (72.5%) patients were delivered vaginally and 11 (27.5%) by cesarean section. Thirty-five (87.5%) patients were receiving mother’s milk and 5 (12.5%) mother’s milk supplemented by formula. No subject was on formula alone. Testis volume was measured as 1 mL in 6 male patients (30%), 2 mL in 12 (60%) and 3 mL in 2 (10%). Laboratory values of the breast enlargement cases are shown in Table 1. Normal ranges accepted for the biochemical parameters were: FSH male: 0.2-4.1 mIU/mL, female: 0.2-14.2 mIU/mL, LH: 0.0-7.0 mIU/mL, estradiol male: 0.1-32 pg/mL, female: 0.1-50 pg/mL, testosterone total, male: 0.075-4 ng/mL, female: 0.0-0.64 ng/mL, prolactin 30-495 ng/dL, fT4: 0.6-1.4 ng/dL, thyroid TSH: 0.6-7 µIU/mL.

Mean age in the control group was 21.6±4.2 days (14-28 days), body weight 3.9±0.6 kg (3-5.5 kg) and length was 51.5±2.0 cm (46-55 cm). Twenty-three (57.5%) of the cases in the control group were delivered vaginally and 17 (42.5%) by cesarean section. Thirty-three (82.5%) were receiving mother’s milk and 7 (17.5%) were on mother’s milk and formula. No babies were on formula alone. Testis volume in male subjects was 1 mL in 10 (50%) and 2 mL in 10 (50%). Laboratory values for the control group cases are shown in Table 1.

Minimum, maximum, mean and standard deviation kisspeptin values as well as p-values by sex and breast enlargement are shown in Table 2.

There was a significant difference in kisspeptin levels between the study and control groups (p=0.039). These groups also differed significantly in terms of prolactin concentrations (p=0.006). No significant correlation was determined of breast enlargement with body weight (p=0.319), length (p=0.228), body mass index (p=0.683), postnatal age (p=0.283), LH (p=0.309), testosterone (p=0.619), fT4 (p=0.968), TSH (p=0.419), FSH (p=0.453), estradiol (p=0.817) and FSH/LH ratio; breast enlargement was not associated with type of delivery (p=0.16) or type of nutrition (p=0.531) as well.

A significant correlation was determined between kisspeptin levels and LH (p=0.05, r=-0.312). Correlation analysis data in the breast enlargement and control groups are shown in Table 3.

## DISCUSSION

Minipuberty occurs in all infants in the newborn period. The release of kisspeptin in the neonatal period may possibly be related to the activation of the hypothalamic-pituitary-gonadal axis in this period of life. Various studies have examined kisspeptin levels in children and adults, but our scan of the literature revealed no studies investigating plasma kisspeptin levels in infants with breast enlargement in the newborn period.

Studies have reported that kisspeptin levels in the circulation are sexually dimorphic in humans, with kisspeptin levels in females being significantly higher than in males (9,10,11,12,13,14). In our study also, mean kisspeptin levels in infants without breast enlargement were slightly higher in the girls. Mean kisspeptin levels in the female infants with breast enlargement were also significantly higher as compared to the boys.

Plasma kisspeptin levels in different studies have been reported at 3.0±1.2 ng/dL in children aged 3-8 years (8), 40.9±3.3 pmol/L in children aged 2-18 (14) and 6.2±2.0 ng/mL in adults aged 18-40 (15). All these levels are higher than the levels in our study. This difference may be attributed to our patients being in the newborn period.

Girls and boys with breast enlargement weighed more than girls and boys in the control group and also had relatively higher kisspeptin levels. While the findings suggest that mean plasma kisspeptin levels rise with mean body weight, no significant correlation was determined between body weight/body mass index and plasma kisspeptin levels. One previous study, however, reported significantly higher kisspeptin levels in prepubertal obese girls compared to healthy prepubertal girls (12).

Kisspeptin has also been shown to be a powerful LH secretion stimulator and to lead to an increase in LH levels in males and females (2,16,17,18). Similarly, in girls, a positive correlation has been determined between serum kisspeptin levels and development of puberty and also between kisspeptin levels and bone age, LH and LH/FSH ratio (6). Another study reported that kisspeptin increased FSH release seven times more compared with LH (17). According to another study, in order to establish a significant increase in serum FSH, a dose 100 times higher than that required to increase LH needed to be given (19). In our study, a significant correlation was found between plasma kisspeptin and LH levels (p=0.050).

Prolactin is one of the major hormones in breast growth (20). Similar to our findings, kisspeptin levels were reported to show a significant correlation with prolactin levels in cases of premature thelarche (8). Studies have also shown that intraventricular injection of kisspeptin-10 increases prolactin levels (21,22). In our study, prolactin levels were higher in infants with breast enlargement than in those without breast enlargement and they were highly significantly correlated (p=0.006).

Akinci et al (8) investigated kisspeptin levels in cases of premature thelarche in 20 female patients aged 3-8 and in 20 healthy controls. Mean kisspeptin level in the premature thelarche group was 3.0±1.2 ng/dL compared to 1.2±0.4 ng/dL in the control group. These levels were also found to be correlated with prolactin levels. The authors suggested that these changes in cases with premature thelarche could be related to a temporary central stimulation. Other studies have also emphasized the importance of kisspeptin in development of central precocious puberty. De Vries et al (7) reported a mean plasma kisspeptin level of 14.6±10.2 pmol/L in 31 girls with central precocious puberty compared to 8.4±1.0 pmol/L in 14 prepubertal girls. One study from Korea compared 30 female patients with central precocious puberty and 30 healthy female controls and found a significant difference (6). Mean kisspeptin level was 4.6±1.8 pmol/L in the girls with central precocious puberty and 2.2±1.5 pmol/L in the control group. The authors emphasized the role of kisspeptin in the onset of puberty and suggested that kisspeptin could be used as a marker of central precocious puberty. Demirbilek et al (23) investigated the role of kisspeptin in central precocious puberty in Turkey. Twenty-eight girls with central precocious puberty were given pubertal suppression therapy for 6 months. At the end of that period, kisspeptin levels in the patient group were compared with those of the 13-year-old control group. In the patient group, initial kisspeptin levels were 10.2±2.6 pg/mL and after 6-month suppression therapy, final kisspeptin levels were 7.3±1.3 pg/mL. Control group kisspeptin levels were 8.6±1.5 pg/mL. These results indicated that the pubertal suppression therapy was effective. These authors proposed that central precocious puberty could be monitored using kisspeptin levels (23). In our study, a significant difference was found in mean plasma kisspeptin levels between the infants with breast enlargement and the control group (p=0.039). Considering the minipuberty occurring in the newborn period, the release of kisspeptin in these infants may be regarded also to be the result of central activation. The higher kisspeptin levels in newborn infants with breast enlargement as compared to the control group is an important finding showing that the hypothalamic-pituitary-gonadal axis is more active in these infants.

In conclusion, the results of this study suggest that plasma kisspeptin and serum prolactin levels may be involved in the physiopathology of breast enlargement in newborns.

## Figures and Tables

**Table 1:**
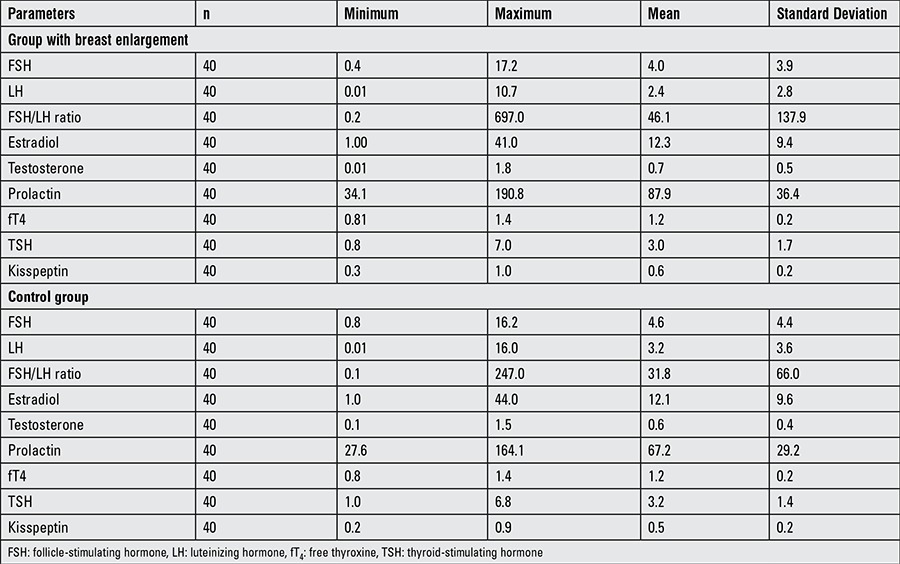
Laboratory values in infants with breast enlargement and in the control group

**Table 2:**
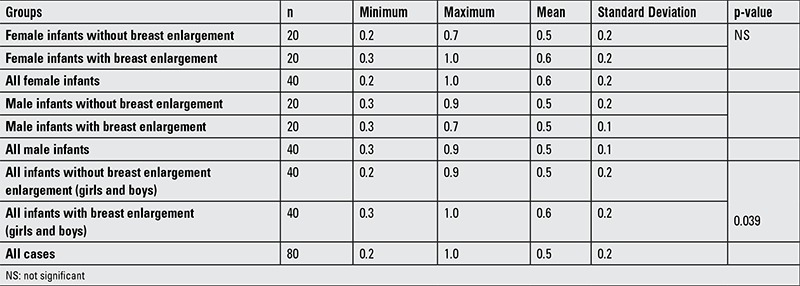
Kisspeptin levels according to sex and breast enlargement

**Table 3:**
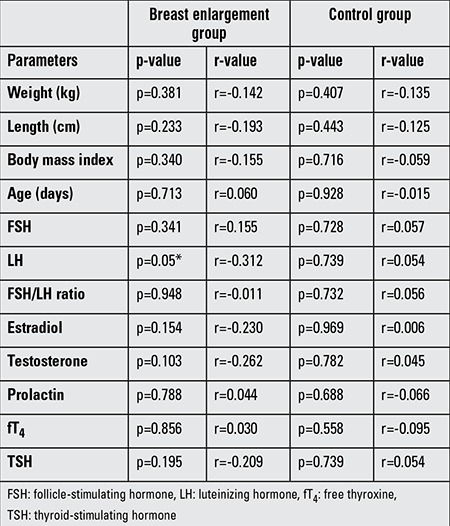
Correlation analysis of kisspeptin and other parameters in the group with breast enlargement group and in the controls
